# Why do open-label placebos work? A randomized controlled trial of an open-label placebo induction with and without extended information about the placebo effect in allergic rhinitis

**DOI:** 10.1371/journal.pone.0192758

**Published:** 2018-03-07

**Authors:** Michael Schaefer, Tamay Sahin, Benjamin Berstecher

**Affiliations:** Medical School Berlin, Calandrellistr, Berlin, Germany; TNO, NETHERLANDS

## Abstract

**Background:**

Several studies demonstrated that placebo treatment may have a significant impact on many different symptoms. While in the traditional view concealment of the placebo is essential, recent studies report intriguing evidence that placebos may work even without deception. For example, it has been demonstrated that open-label placebos can improve symptoms in allergic rhinitis. However, the mechanisms of how placebos without concealment work remain unknown.

**Trial design:**

In order to examine expectancy effects we conducted a randomized controlled trial (N = 46), in which patients with allergic symptoms received either placebos without deception or no pills at all. In half of those patients we induced positive expectations about the placebo effect. After two weeks we tested whether symptoms and quality of life had changed.

**Results:**

Results revealed that open-label placebos improved allergic symptoms more than the control group. Inducing positive expectations had no effects on the improvement of allergic symptoms (the primary and more objective outcome), but on mental sum scores of the quality of life questionnaire.

**Conclusions:**

Placebos without deception can improve symptoms in allergic rhinitis. Positive expectations do not contribute to the efficacy of open-label placebos, but seem to have an effect on more global and subjective well-being (mental or emotional quality of life).

**Clinical trial registration number:**

German Clinical Trials Register, DRKS00012303

## Introduction

The word allergy was first used by the Austrian scientist Clemens von Pirquet in 1906 [1]. Allergic diseases are defined as conditions caused by hypersensitivity of the immune system to something in the environment that in general causes no problems in most people. Allergies are very common in the western world; about 20% of people in the developed world describe symptoms of allergic rhinitis [2]. There are a lot of medications available to successfully block allergy reactions. However, it has also been suggested that psychological factors can be important at least for some allergy types. In fact, the placebo effect is high in allergic rhinitis [3–5].

Numerous studies have demonstrated that placebo treatment can have a significant impact for a wide variety of symptoms [6]. Nevertheless, placebo treatment may cause severe practical and ethical problems as deception is thought to be necessary and would therefore undermine informed consent and trust [7].

Recent studies question whether deception or concealment is necessary to elicit placebo effects. Kaptchuk et al. conducted a two-group randomized controlled study including patients with irritable bowel syndrome [8]. Patients were randomized to either open-label placebo pills or no-treatment controls. After three weeks the authors found significantly higher mean global improvement scores, reduced symptom severity, and better quality of life scores for the open-label placebo group. Thus, the patients demonstrated a placebo effect—although they knew that they were taken a placebo. Similarly, a recent study showed that open-label placebos also work to reduce symptoms in allergic rhinitis. A two-group randomized controlled pilot study included patients with allergic rhinitis. One group received placebos without deception, the other no pills. Both groups were matched with respect to patient-provider interaction. After two weeks results revealed that open-label placebos improve allergic symptoms better than the control group [9]. Other studies in chronic low back pain and episodic migraine had similar outcomes [10, 11].

While there is an increasing body of evidence that open-label placebos are effective for improving symptoms [8–14], it still remains unclear how placebos without deception might influence patients. The effects of placebos have been explained by associative learning (e.g., classical conditioning and other non-conscious associative learning processes) [15–17]. This mechanism has also been suggested to explain the mechanisms in open-label placebos [16, 17]. In addition, placebo effects are related to the social interaction with healthcare practitioners. Given that most of the open-label placebo studies tried to parallel the amount of healthcare-provider interaction, it seems unlikely that the open-label placebo effects may be explained by this mechanism. Furthermore, another reason why placebos work seems to be conscious expectations [18]. This mechanism has also been discussed to explain the open-label placebo effect [19]. Although patients when receiving open-label placebos do not have the same level of conscious expectations as deceptive placebos, open-label placebos are usually combined with positive suggestions.

For example, participants often were briefed in a typical way [8, 9, 13]. They were explained that although placebos are inactive substances and contain no medication, placebo effects may still be powerful. The effect was explained to them by pointing out that the body may automatically respond to taking placebo pills, like Pavlov’s dogs that salivated when they heard the bell. In addition, they were told that a positive attitude may be helpful for the placebo effect, but is not necessary. Finally, they were told that those participants who were in the placebo group needed to take the placebos faithfully.

Does this detailed information about powerful effects of placebos raise so many positive expectations that they can account for the open-label effects? In order to examine this question the current study investigates the improvement of allergic symptoms by open-label placebos depending on positive expectancies (detailed information of the power of placebos). Thus, the present study aims to examine the role of expectations for the open-label placebo effect.

## Methods

### Participants

All patients provided written informed consent. The study was done in accordance with the Declaration of Helsinki and approved by the ethical board of the German Psychological Association.

Participants were recruited from fliers in a local university and via social media. Inclusion criteria was age (between 18 and 60 years) and allergic rhinitis diagnosed by a physician. Only participants taking medication were included. Participants were asked not to change medications or dosages during the study. Exclusion criteria were pregnancy, diabetes and any psychiatric or neurological diseases (e.g., major depression disorder).

The data were collected at the Medical School Berlin, Germany. Data of our previous study [9] were not included in this trial. In addition, none of the patients participating in the previous trial were invited for this second study.

**Fig 1:**
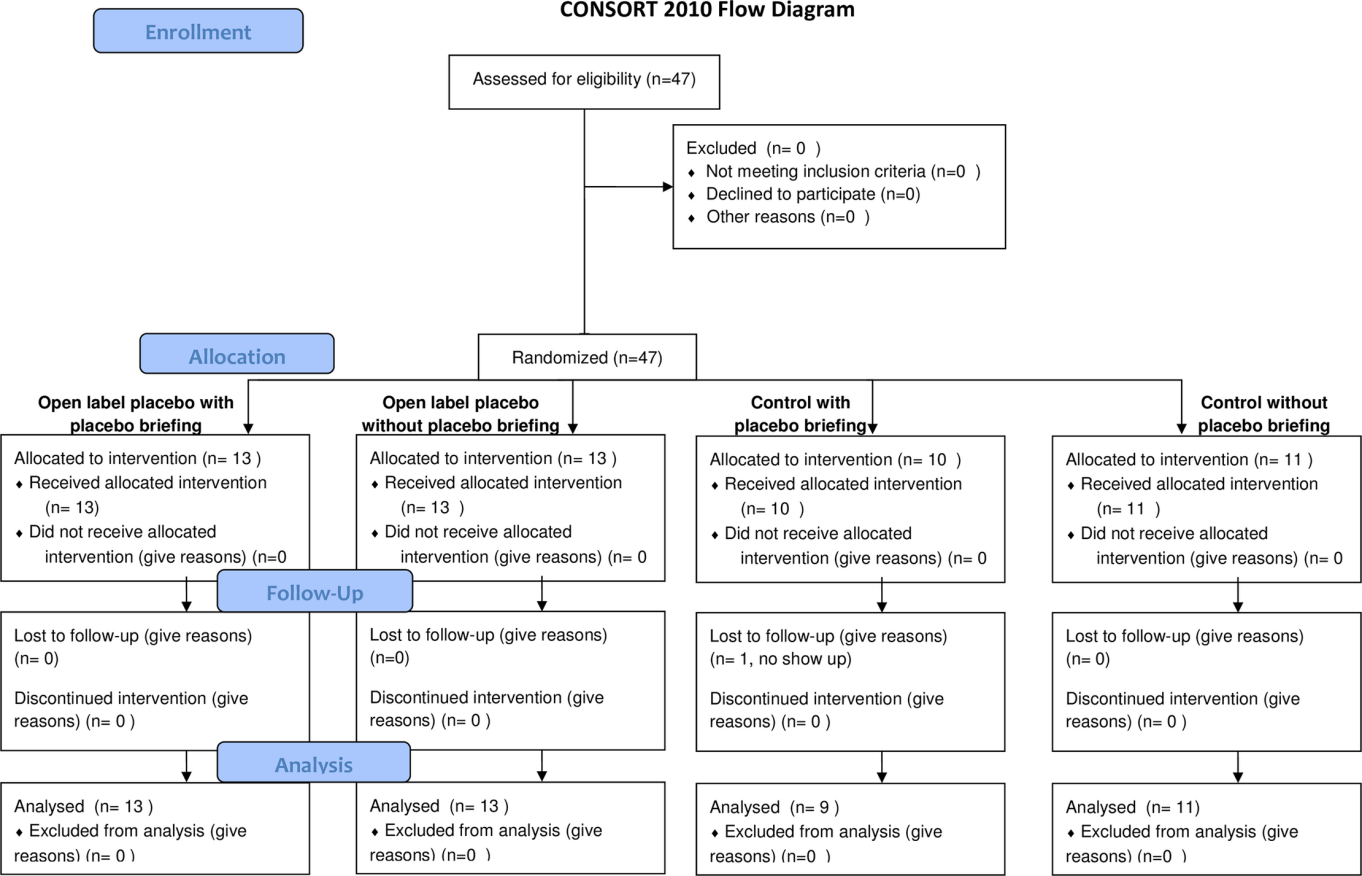
Flow diagram of patient’s enrollment.

We enrolled a total of 47 participants during springtime and early summer 2016 (data acquisition for this pilot study was stopped after ending of main pollen period). One participant did not show up for the second measurement and was excluded prior to data analysis, resulting in 46 patients for the follow-up. The resulting groups included 13 participants for the group with placebos and positive expectancy briefing, 9 for the control group with positive expectancy briefing, 13 for the group with placebos and without positive expectancy briefing, and 11 for the control group without positive expectancy briefing (Fig 1 and S1 Dataset). Demographic and clinical characteristics are displayed in Table 1. All participants were Germans.

**Table 1:** Demographic and clinical characteristics for different groups.

Variable	Open label placebo with placebo briefing	Open label placebo without placebo briefing	Control with placebo briefing	Control without placebo briefing
N	13	13	9	11
Age (mean ±SD in years)	25 ±9	23 ±3	26 ±10	26 ±4
Females / males	9 / 4	9 / 4	9 / 0	10 /1
Taking Cetiricine	4	5	4	2
Taking Loratandin	3	1	1	1
Taking others	6	7	4	8
Marital status (married or partnership)	9	3	5	7
Participants without employment	3	3	4	3

### Design

We conducted a two week randomized controlled trial (RCT) including 46 patients comparing open-label placebo to no-treatment controls (with and without information on how powerful placebos are) (clinical trials Nr. DRKS00012303, registered 4 / 2017) (trial was registered after participant recruitment began in order in order to cover the pollen time). The authors confirm that all ongoing and related trials for this drug/intervention are registered.

Sample size was determined following previous studies [8, 9]. Based on a desired power of .80, an alpha error probability of .05 and an estimated effect size of d = 0.9, the required number of participants was a priori set to n = 14 per condition. Given that this is a pilot study and data collection was limited due to weather (pollen) conditions, the number of patients enrolled in this study was 47 (see Fig 1).

We used a two-factorial experimental design. The first factor was open-label placebos relative to no pills. The second factor detailed information on placebos (positive expectancy) relative to no information. We examined the no-pills group with regard to beneficial effects due to detailed information on placebos, too, in order to control for spontaneous improvement.

Thus, patients were randomized in four groups (open-label placebo with positive expectancies, open-label placebo without positive expectancies, no pills with positive expectancies, no pills without positive expectancies). Patient-provider interaction and amount of contact time was held similar for all groups. After two weeks we tested whether symptoms and quality of life had changed.

### Procedure

Before assigning each of the participants randomly to one of the four groups, all subjects were informed that this study was about psychophysical interactions during the treatment of allergies. They were told that they had to use an allergic diary in which they had to note their individual allergic complaints at the end of each day. Furthermore, they were told that they were assigned to different groups. One group would receive placebos, which they should take twice each day. Placebos were explained as containing no medications, similar to a sugar pill. Another group would get no pills.

Then the participants were randomized in two groups (by choosing a sealed opaque envelope). Half of the participants received additional information on the power of placebos (group with positive expectations). They were told that placebos are inactive substances and that they contain no medications. Participants were further told that although placebos contain no medication, placebo effects may still be powerful. The effect was explained to them by pointing out that the body may automatically respond to taking placebo pills, like Pavlov’s dogs that salivated when they heard the bell. In addition, they were told that a positive attitude may be helpful for the placebo effect, but is not necessary. Last, they were told that those participants who were in the placebo group needed to take the placebos faithfully. These four statements are identical to the instruction used in previous studies on open-label placebos [8, 9, 13]. The other half of the participants received no such additional information on the power of placebos (group with no positive expectancies).

After this briefing the participants were asked to complete a set of questionnaires in order to assess their allergy and their quality of life. First, assessment of the participants contained a self-developed questionnaire about their allergic symptoms (identical to [9]). Participants were asked to assess their allergic symptoms by using a questionnaire describing different symptoms (itching, prickling, or burning feelings in the nose; constipated or running nose; impaired sense of smell or taste; sneezing fits; feeling like having a cold; itching or irritated skin; eczema on the skin; itching, burning or red eyes; billowed eyelids; itching, prickling or scratching feelings in the throat; sore throat; burning feeling or mucus in the mouth or throat; billowed mucosa; breathlessness; cough; headaches; feelings of exhaustion; lack of concentration; tiredness; disorders of the gastrointestinal tract). Participants indicated their response on a seven-point scale ranging from ‘never’ (1) to ‘always’ (7). For further analysis we calculated a composite score including all symptoms. This primary outcome measure was identical to our previous study [9].

Second, we used the SF-36, a German version of the health survey developed by Ware and Sherbourne in order to examine quality of life [20]. This instrument assesses the quality of life with respect to the perception of the health both for patients and healthy people. It includes one multi-item scale that assesses different health concepts such as limitations in physical activities because of health problems, limitations in social activities because of physical or emotional problems, limitations in usual role activities because of physical health problems, bodily pain, general mental health (psychological distress and well-being), limitations in usual role activities because of emotional problems, vitality (energy and fatigue), and general health perceptions. The survey is constructed for self-administration. None of the participants stated to have any differences to complete the questionnaires.

After completing these questionnaires all participants were randomized by choosing a sealed opaque envelope in which there was the assignment of being either in the placebo group or in the control group. Participants in the placebo group then received a white tube containing 28 placebo pills. The tube was labeled with logo of the local university and the following information: “Placebo pills, take one in the morning and one before night, for 14 days”. The placebo pills were white, round, about 4 mm and contained sugar, wheat- and cornstarch, and glucose syrup. The participants were told to swallow the pills, not to chew or suck them. They were told to come back in 14 days for the second appointment.

Participants in the control group (with and without detailed briefing) received no pills at all. They were reminded of the importance of the control condition for a successful study and asked for not missing the second appointment. Furthermore, all participants were reminded to note their individual allergic complaints at the end of each day in the allergic diary.

Participants of all four groups were asked not to change medications or dosages during the study. In addition, they were asked to refrain from making any changes in their life-style patterns.

During the second appointment subjects underwent again the assessment of their allergic symptoms and also of their state of health. The experimenter was blind to treatment assignments. Last, participants were asked about possible feelings of disappointment for those of them who have been assigned to the control group.

### Statistical analysis

Statistical analysis of the results included ANOVAs of allergic symptoms before and after the treatment for both groups (mixed 3-factorial ANOVA with the factors expectancy (information vs. no detailed information on placebos), group (open-label placebos vs. no placebos) and time (first vs. second measurement). The healthy survey (SF-36) results in two different outcome parameters: The physical sum score reports quality of life with respect to the body state, whereas the mental sum score describes the psychic dimension of subjective well-being (mental or emotional quality of life). Both measures went into separate ANOVAs analogue to the previous procedure.

Changes in allergic symptoms (composite score) and quality of life (physical or mental sum score), respectively, were analyzed via paired sample t tests. We report Cohen’s D to provide information about the power of possible effects of these comparisons.

For statistical analysis we used the SPSS software package (IBM Corp., Armonk, NY, USA). A p value of < 0.05 was considered as the level of significance.

## Results

We first examined possible effects on allergic symptoms (composite score). Results of an ANOVA (factors time: pre/post treatment, placebo pills: yes/no, expectancy: detailed information on placebos / no detailed information) revealed a significant interaction of the factors time and placebo (F (1,42) = 5.42, p = 0.02; η^2^ = 0.11) (see Table 2). Post hoc t-tests demonstrated an improvement from the first to the second measurement only when participants received open-label placebos (open-label placebo group, mean ±; pre: 3.27 ±0.81, post: 2.66 ±0.75, difference score: 0.61; control group; pre: 3.36 ±1.03, post: 3.32 ±1.02, difference score: 0.05; t(44) = 2.40, p = 0.02; Cohen’s d = 0.74).

**Table 2:** Results of the ANOVA (factors time: Pre/post treatment, placebo pills: Yes/no, expectancy briefing: Detailed information on placebos / no detailed information; depending variables: Composite symptom scores, physical and mental sum scores of the SF-36) significant results in bold, no other significant results were found).

depending variable	factors	F	p
composite symptom scores	main effect time	8.38	**0.006**
	interaction time and placebo	5.42	**0.02**
	interaction expectancy briefing and placebo	1.42	0.24
physical sum scores (SF-36)	main effect time	0.13	0.72
	interaction time and placebo	0.007	0.93
	interaction expectancy briefing and placebo	0.49	0.51
mental sum scores (SF-36) (change scor.)	main effect placebo	0.57	0.45
	main effect expectancy briefing	8.45	**0.006**
	interaction expectancy briefing and placebo	1.50	0.23

**Fig 2:**
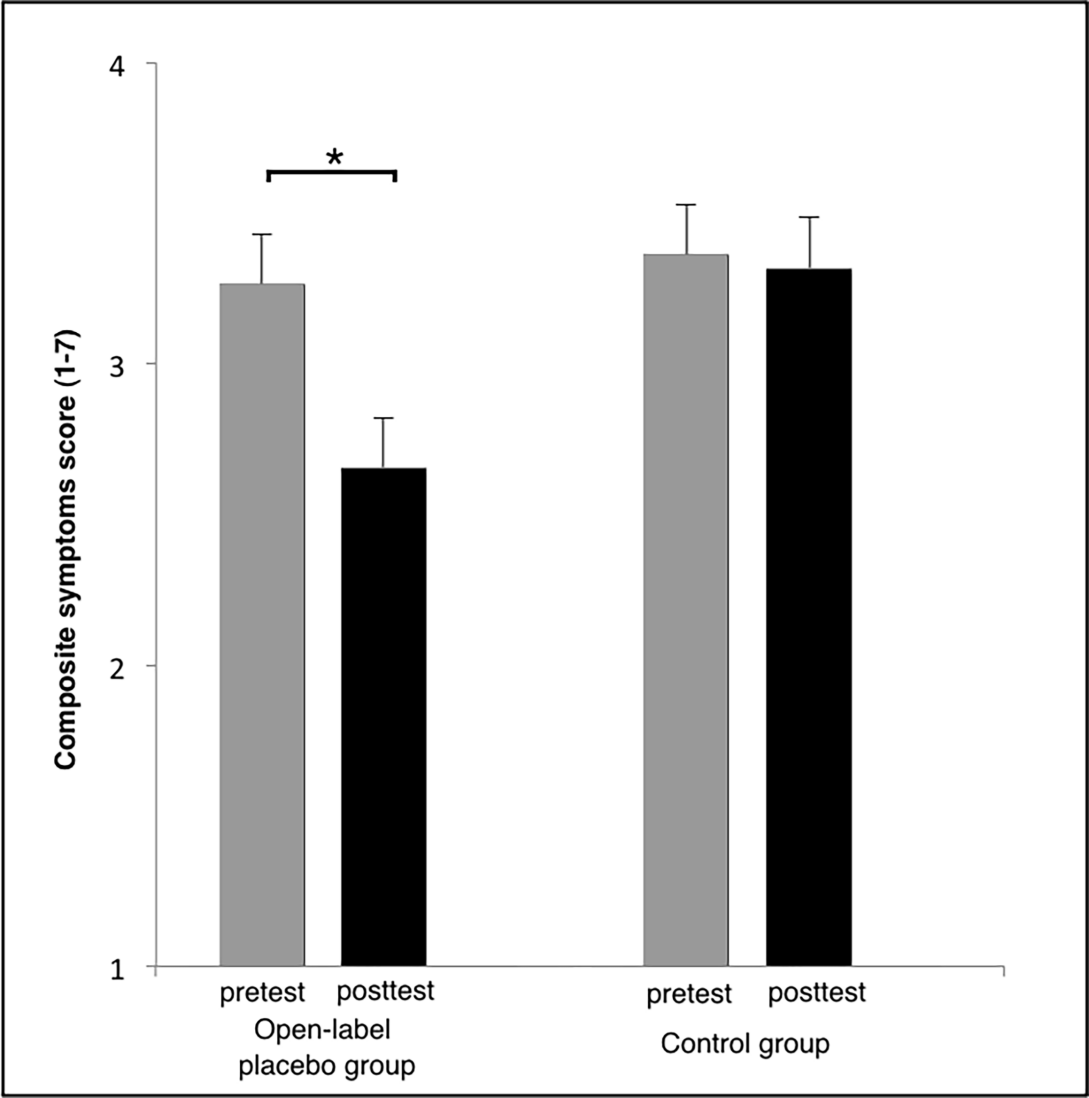
Changes of allergic symptoms for open-label placebo and control group (mean and standard error). Patients indicated their response on a seven-point scale ranging from ‘never’ (1) to ‘always’ (7) (composite score of all symptoms). Results demonstrate significantly stronger improvement for the open-label placebo group relative to the control group. * p = 0.02.

**Fig 3:**
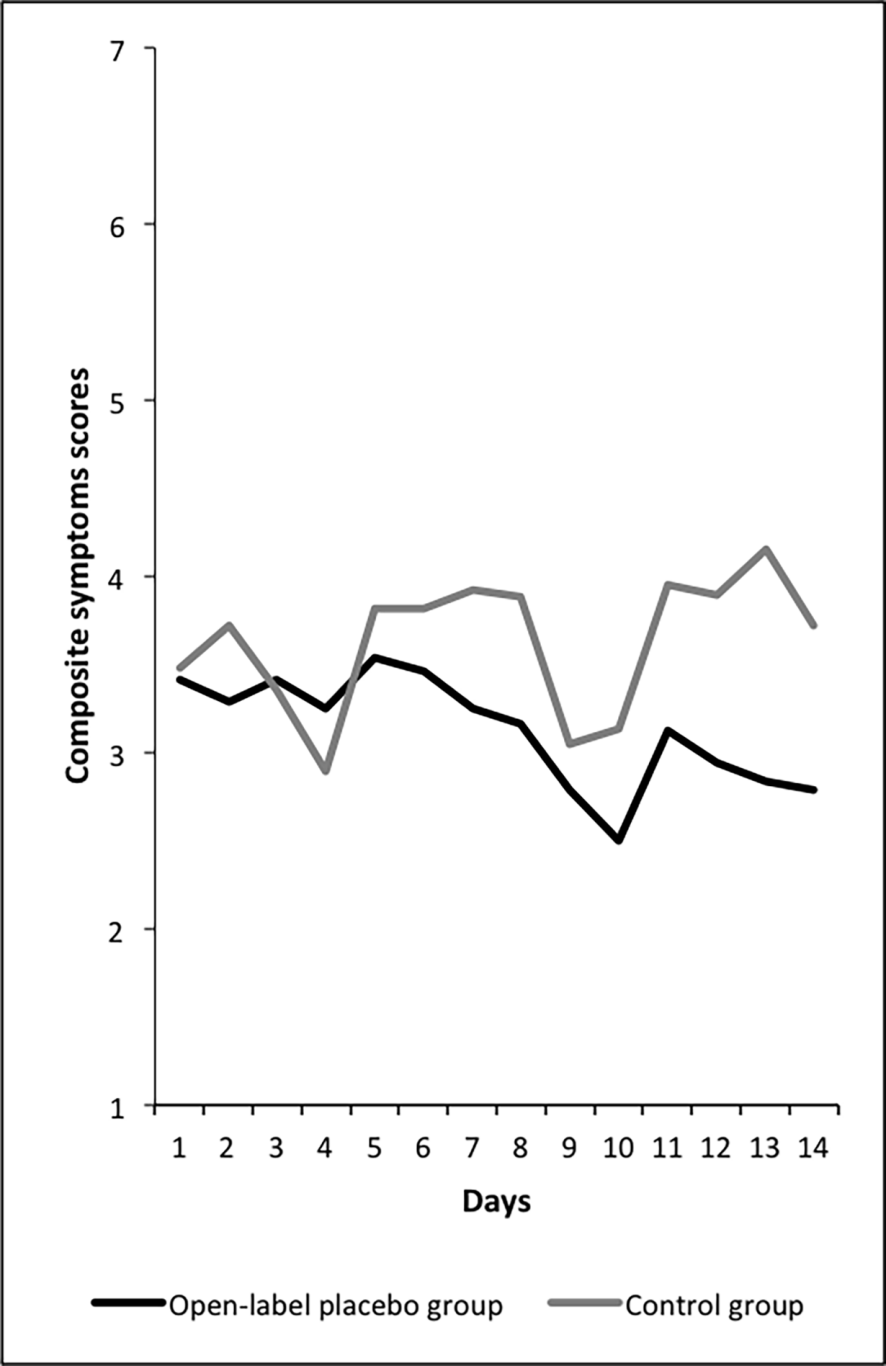
Data of the allergic diary demonstrate improvement of the open-label placebo group over time.

Thus, the open-label placebo effect for allergic rhinitis could be replicated (see Fig 2 illustrating this effect and Table 3 depicting results for every single group). The data of the allergic diary demonstrates that the effect is taking place only few days after taking the open-label placebo (see Fig 3).

**Table 3:** Treatment outcomes (pre, post, and change scores).

	Open-label placebo with Placebo- Briefing	Open-label placebo withoutPlacebo- Briefing	Control with Placebo- briefing	Control without Placebo- briefing	Open-label placebo	Control
Quality of Life (SF-36): physical sum score (mean ±SD)	pre: 53.19 ±8.09 post: 54.16 ±3.44 change: 0.97 ±6.25	pre: 53.13 ±6.72 post: 51.57 ±8.52 change: -1.57 ±6.95	pre: 50.12 ±09.95 post: 50.39 ±6.69 change: 0.27 ±5.86	pre: 52.05 ±7.53 post: 50.82 ±7.02 change: -1.23 ±9.54	pre: 53.16 ±7.29 post: 52.86 ±6.50 change: -0.30 ±6.60	pre: 51.18 ±8.52 post: 50.63 ±6.70 change: -0.56 ±7.93
Quality of Life (SF-36): mental sum score (mean ±SD)	pre: 44.08 ±8.30 post: 45.50 ±6.97 change: 1.42 ±7.90	pre: 41.01 ±10.40 post: 41.34 ±11.56 change: 0.33 ±6.47	pre: 39.22 ±10.95 post: 42.16 ±11.19 change: 2.94 ±7.23	pre: 47.75 ±6.11 post: 46.01 ±6.98 change: -1.73 ±6.49	pre: 42.54 ±9.35 post: 43.42 ±9.59 change: 0.87 ±7.10	pre: 43.91 ±9.44 post: 44.28 ±9.07 change: 0.38 ±6.79
Allergic Symptoms (composite score, mean ±SD)	pre: 3.31 ±0.70 post:2.53 ±0.63 change: 0.79 ±0.65	pre: 3.22 ±0.94 post: 2.79 ±0.86 change: 0.43 ±0.83	pre: 3.75 ±0.91 post: 3.47 ±0.94 change: 0.28 ±0.81	pre: 3.05 ±1.06 post: 3.19 ±1.11 change: -0.15 ±0.84	pre: 3.27 ±0.81 post: 2.66 ±0.75 change: 0.61 ±0.75	pre: 3.36 ±1.03 post: 3.32 ±1.02 change: 0.05 ±0.83
	Open-label placebo with Placebo- Briefing	Open-label placebo without Placebo- Briefing	Control with Placebo- briefing	Control without Placebo- briefing	Open-label placebo	Control
Quality of Life (SF-36): physical sum score (mean ±SD)	pre: 53.19 ±8.09 post: 54.16 ±3.44 change: 0.97 ±6.25	pre: 53.13 ±6.72 post: 51.57 ±8.52 change: -1.57 ±6.95	pre: 50.12 ±09.95 post: 50.39 ±6.69 change: 0.27 ±5.85	pre: 52.05 ±7.53 post: 50.82 ±7.02 change: -1.23 ±9.54	pre: 53.16 ±7.29 post: 52.86 ±6.50 change: -0.30 ±6.60	pre: 51.18 ±8.52 post: 50.63 ±6.70 change: -0.56 ±7.93
Quality of Life (SF-36): mental sum score (mean ±SD)	pre: 44.08 ±8.30 post: 45.50 ±6.97 change: 1.42 ±7.90	pre: 41.01 ±10.40 post: 41.34 ±11.56 change: 0.33 ±6.47	pre: 39.22 ±10.95 post: 42.16 ±11.19 change: 2.94 ±6.59	pre: 47.75 ±6.11 post: 46.01 ±6.98 change: -1.73 ±6.49	pre: 42.54 ±9.35 post: 43.42 ±9.59 change: 0.87 ±7.10	pre: 43.91 ±9.44 post: 44.28 ±9.07 change: 0.38 ±6.79
Allergic Symptoms (composite score, mean ±SD)	pre: 3.31 ±0.70 post:2.53 ±0.63 change: 0.79 ±0.65	pre: 3.22 ±0.94 post: 2.79 ±0.86 change: 0.43 ±0.83	pre: 3.75 ±0.91 post: 3.47 ±0.94 change: 0.28 ±0.81	pre: 3.05 ±1.06 post: 3.19 ±1.11 change: -0.15 ±0.84	pre: 3.27 ±0.81 post: 2.66 ±0.75 change: 0.61 ±0.75	pre: 3.36 ±1.03 post: 3.32 ±1.02 change: 0.05 ±0.83

Importantly, there was no interaction between the factors expectancy, time, and placebo (p = 0.89) or expectancy and placebo (p = 0.24). The severity of the symptoms before the experiment was not different between the groups (with respect to placebo groups: p = 0.72; with respect to information groups: p = 0.19). Thus, open-label placebos improved symptoms in allergic rhinitis independently of detailed information about placebos.

Furthermore, we found a main effect for time, indicating that symptoms improved over time irrespective of treatments (F (1,42) = 8.38, p = 0.006). This general effect may be explained by an improved pollen situation at the time of the second measurement.

**Fig 4:**
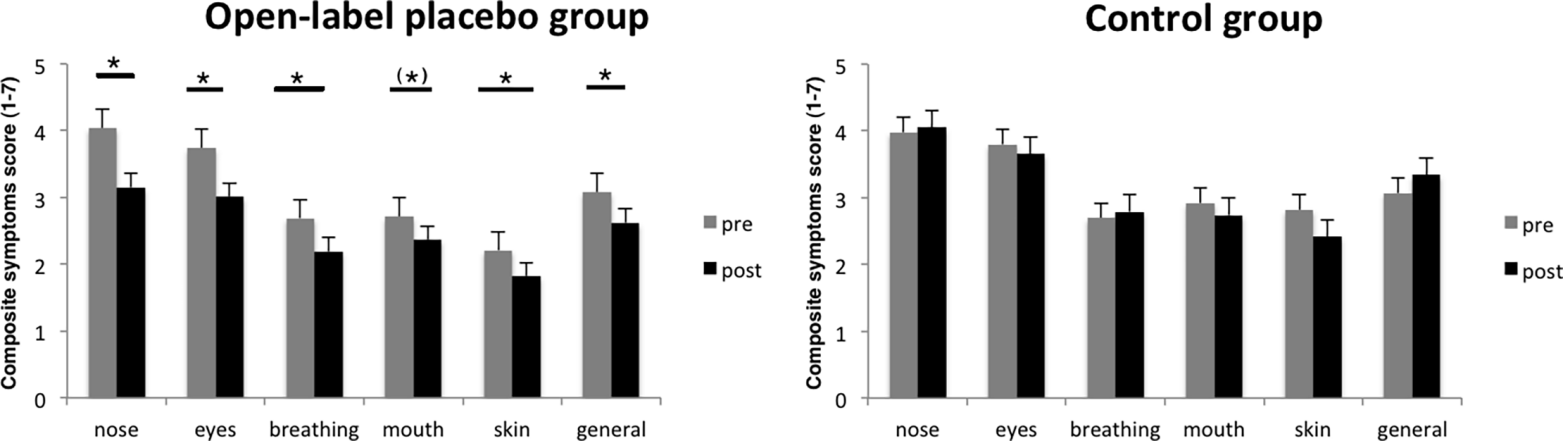
Changes of allergic symptoms for open-label placebo and control group for single symptom groups (mean and standard error). Results show significant improvements for the open-label placebo group but not for the control group. * p < 0.05, (*) p = 0.06.

In order to investigate treatment effects on symptoms more in detail, we created symptom groups related to discomforts of the nose, breathing system, eyes, mouth, skin, and general feeling (composite scores for nose: itching, prickling, or burning feelings in the nose, constipated or running nose, impaired sense of smell, sneezing fits, feeling like having a cold; composite scores for breathing system: breathlessness, cough; composite scores for eyes: itching, burning or red eyes, billowed eyelids; composite scores for mouth: itching, prickling or scratching feelings in the throat, sore throat, burning feeling or mucus in the mouth or throat, billowed mucosa, impaired sense of taste; composite scores for skin: itching or irritated skin, eczema on the skin; composite scores for general feeling: headaches, feelings of exhaustion, lack of concentration, tiredness, disorders of the gastrointestinal tract). For all of these symptoms we found significant improvements in the open-label placebo group but no effects for the control group (Fig 4).

**Fig 5:**
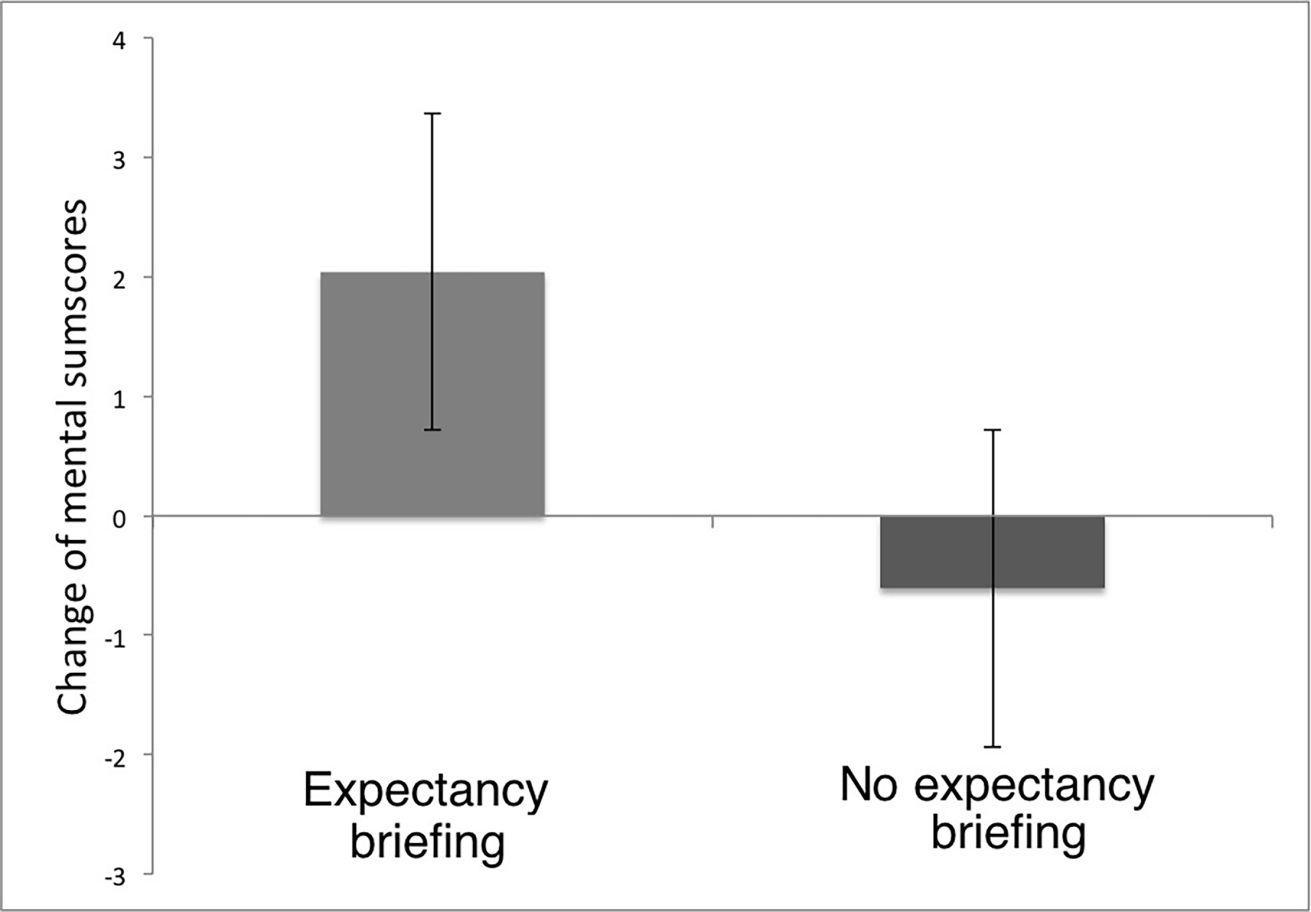
Change of mental or emotional quality of life (mental sum score of the SF-36) when receiving detailed information on placebos (raising positive expectancies) compared with no detailed information about the power of placebos. Results revealed stronger improvement of mental or emotional quality of life when being told that placebos are powerful (irrespective of receiving open-label placebos).

We then investigated effects on quality of life (mental and physical sum scores of the SF-36). An ANOVA (factors time, placebo, expectancy) showed no effects on physical or mental sum score of the SF-36 (physical sum score before was not different, p = 0.76). Since mental sum score scores were different prior to the intervention (t(44) = 36.09; p = 0.001), we calculated an ANOVA with difference scores as dependent variable and mental sum scores scores prior to the study as a covariate. Results showed no effects for placebos on mental sum score (all p > 0.10, see Table 2). In contrast, we found a significant effect of expectancy on mental sum score (difference scores, detailed information: 2.04 ±7.27; no detailed information: -0.61 ±6.42; F(1,41) = 8.45, p = 0.006; η^2^ = 0.21). There was no significant interaction of expectancy with placebos on mental sum score (p = 0.23). Hence, detailed information (positive expectancies) on the way placebos work influenced mental sum scores of the SF-36, independent of taking open-label placebos (see Fig 5 illustrating this main effect and Table 3 depicting results for every single group).

Last, we asked the participants in the control group if they were disappointed not to be in the placebo group. None of the participants claimed to be disappointed. Furthermore, none of our participants reported any adverse effects due to the placebos.

## Discussion

Several studies demonstrated that placebos without concealment reduce symptoms in different diseases [12–14]. Our recent study suggested that open-label placebos may also reduce symptoms in allergic rhinitis. This study aimed to test if expectancy is a crucial factor for open-label placebo effects in allergic rhinitis. Positive expectancy was manipulated by giving detailed information on the power of placebos. Our results demonstrated that open-label placebos improve symptoms of allergic rhinitis better than a control group with comparable patient-adviser contact. This effect was not related to positive expectancies (level of detailed information on the beneficial effects of placebos). In contrast, expectancies did affect the mental sum score of the SF-36, independent of the open-label placebo effect.

Previous research showed that open-label placebos successfully reduce symptoms, for example, in irritable bowel syndrome and other diseases [12–14]. Our recent pilot study suggested that placebos without deception may also improve symptoms in allergic rhinitis [9]. The present study confirm these results, open-label placebos improved symptoms of allergic rhinitis better than a control group with comparable patient-adviser contact. This improvement was not restricted to any specific symptom group, such as symptoms related to the nose or the eyes. Thus, all symptoms in allergic rhinitis seem to benefit from open-label placebo treatment.

Open-label placebo studies usually inform the patients always in the same way. Four statements are given to the patients, explaining to them why placebo effects may still be powerful although they not contain any medication. Is this crucial to evoke effects in an open-label placebo study or could we perhaps pass this information? In order to test a possible interaction of the open-label placebo effect with expectancy (detailed information of how placebos work), we manipulated this information in our study. Results demonstrated that the open-label placebo effect was not related to the level of information on placebos we gave to them. Furthermore, level of information on placebos alone did not affect improvement of symptoms. Thus, the effect of taking placebos cannot be accounted to the level of information on placebos in general, which may have raised expectancies that placebos are effective.

Hence, although still other expectancies may be evoked when participating in a placebo study, these results make it unlikely that the results of open-label placebos are simply caused by expectancy mechanisms. Our findings are also supported by an experiment in episodic migraine pain that seemingly inadvertently compared open-label placebo without expectations producing a 30% reduction in pain compared to no-treatment controls [11]. Future research is needed in order to further examine how open-label placebos work.

Placebo mechanisms have also been discussed in terms of the theory of embodied cognition [19, 21]. This theory argues that (even abstract) human cognitions can be shaped (or even determined) by the body and it’s interaction with the environment, in particular based on sensorimotor systems. Since this interaction may take place without previous conditioning, embodiment is different from classical conditioning. The theory of embodiment provides many examples by demonstrating psycho-physiological effects [22, 23]. Based on this theory, the body’s response when receiving placebos might also be based on other brain areas than the brain’s reward center [15].

However, a detailed information about the power of placebos may still be very useful because one cannot exclude that in some patients open-label placebos may elicit nocebo responses, e.g., feelings of dizziness or nausea. Thus, although none of our participants reported any adverse effects, information about the beneficial effects of placebos may be important to avoid nocebo responses.

A second outcome measure in this study was quality of life. We did not find any effects of open-label placebos on mental and physical sum scores of the SF-36, thereby confirming previous results [9]. However, we did find a main effect of expectancy on mental sum scores. Thus, more detailed information on how powerful placebos may be (thereby raising positive expectancies) seem to improve the mental dimension of quality of life. This is surprising, because the effect is independent from the placebo factor. Hence, detailed information on how placebos work improves mental sum scores of the SF-36 regardless if we take placebos or not. So why does information on placebos affect patients who do not get any pills? In fact, all of our patients still took their regular medicine and we told them not to change this. Therefore, given that we only included patients taking drugs, everyone in this study was swallowing pills. We assume that the detailed instruction of how mighty placebos may be might have had an impact on the placebo effect of the regular drugs the patients took and a more global and mental or emotional quality of life. Future studies are needed to further test this result.

An alternative explanation of the effect of expectancy on mental sum scores may point to an improved patient-provider-interaction. However, we think this is unlikely because the more detailed information on placebos resulted only in slightly prolonged patient-provider-interaction.

Several limitations of our study should be noted. The sample size of this pilot study is small. Furthermore, the trial duration was rather short and there was no follow-up study. It would have been interesting to have more detailed information about the expectations the patients actually had. Also, a test to verify whether the participants understood that placebos were inert substances may have been included. In addition, the outcome variable here included only self-report data. Future studies should replicate the results with a larger sample set and, for example, also include biological data. Another limitation of the current study is the lack of a comparison of the open-label condition with a closed-label condition. The placebo effects might be different with respect to open- and closed label placebo treatments.

The results of this randomized controlled study underline that placebo without deception may successfully reduce symptoms in allergic rhinitis. In addition, our results suggest that a detailed description of how placebos in general work may not be necessary to induce open-label placebo effects. Nevertheless, detailed information about the beneficial effects of placebo taking may still be important in open-label placebo studies in order to avoid nocebo effects. The outcome of this study also stresses that placebo effects on more global and subjective well-being (mental health) may be influenced just by pointing to some more detailed information of what pills can do. The results may help us to understand how placebos work and should also encourage further studies on placebos without deception.

## Supporting information

S1 Consort 2010 checklist (DOC)

S1 Dataset (XLS)

S1 Study Protocol (DOC)

S1 Study Protocol for Ethical Committee (PDF)
