# Supplementary material for: Why do open-label placebos work? A randomized controlled trial of an open-label placebo induction with and without extended information about the placebo effect in allergic rhinitis
Source: PLoS One. 2018 Mar 7;13(3):e0192758. doi: 10.1371/journal.pone.0192758 (PMC5841659; doi:10.1371/journal.pone.0192758)
Supplement: S1 Study Protocol — (DOC) [file pone.0192758.s003.doc]

Study Protocol: Open-label placebos in allergic rhinitis

Principal Investigator: Michael Schaefer, PhD

Co-Investigators: Tamay Sahin, Benjamin Berstecher

**A. Purpose of Protocol**

This study will test whether placebos without deception can result in clinical improvements in patients with allergic rhinitis.

The study aims to replicate previous findings and also examine possible mechanisms with respect to top-down and bottom-up mechanisms in open-label placebo experiments.

**B. Background for the Study**

Recent research has demonstrated that placebo treatment can have significant impacts for a wide variety of symptoms (Finiss et al., 2010). Unfortunately, in the traditional understanding response to placebos requires deception. This may cause problems with respect to patient-healthcare provider relationship and ethical difficulties. In contrast to this classic view recent research has demonstrated that placebos may work even without concealment. For example, Kaptchuk et al. (2010) conducted a randomized controlled trail including patients with irritable bowel syndrome. Half of the patiens received placebos without deception, the other half served as a control group. The authors found significantly higher mean global improvement scores, reduced symptom severity, and better quality of life scores for the open-placebo group. Similar results have been reported, for example, for patients with major depression (Kelley et al., 2012) and ADHD (Sandler et al., 2008).

Our previous study has demonstrated that open-label placebos also improve

symptoms in patients with allergic rhinitis (Schaefer et al., 2016). However, this pilot study was based on only few patients. Thus, we here want to replicate the results in a larger sample.

Furthermore, the mechanism of open-label placebo effects remains unclear. Are open-label placebo effects based on bottom-up mechanisms, such as classical conditioning? Or are they linked to raised expectancies about the positive effects of placebos in general, hence representing top-down effects? The latter may be related to the detailed information on the power of placebo effects. This information is included in nearly all open-label placebo studies. In order to disentangle top-down and bottom-up effects, we aim to manipulate this information: Half of the participants will be informed about the power of placebos, the other half not.

We expect to replicate the previous findings that open-label placebos improve allergic symptoms more than in a control group with comparable patient-healthcare provider relationships. Furthermore, we assume that the open-label placebo effect is independent of the information how powerful placebos are, thereby suggesting a bottom-up effect.

**C. Aim**

Replication of earlier results if placebos without deception reduce symptoms in patients with allergic rhinitis (Schaefer et al., 2016) and proof of the role of detailed information on how powerful placebos are for this effect.

**D. Hypothesis**

In allergic rhinitis patients, a two weeks course of open-label non-deceptive placebo treatment will be superior to no treatment plus usual care control. In addition, information on the positive effects of placebos will not interact with this open-label placebo effect.

**E. Description of Research Protocol**

Overview: This study aims to test whether non-deceptive administration of placebo in patients with allergic rhinitis will be superior to a control group, and if this effect is independent of detailed information on positive effects of placebos.

Design: 60 patients with allergic rhinitis will be randomized to a two-week course of either non-deceptive placebo pill with detailed information on positive effects of placebos, of non-deceptive placebo pill without detailed information on positive effects of placebos, of no treatment plus usual care with detailed information on positive effects of placebos, and of no treatment plus usual care without detailed information on positive effects of placebos. All groups will receive the same amount of time with the practioner. Participants will be assessed at baseline and at the end of the two weeks.

Design and intervention:

Description of the open-label placebo treatment arms:

After signing the informed consent the patients will be assessed with respect to their allergic symptoms and quality of life. They will be told to note their allergic complaints at the end of each day in an allergic diary. Then they will receive 28 placebos in a white box, labeled with the logo of the university and the following information “Placebo pills (28), take one in the morning and one before night, for 14 days”

Description of the no treatment usual care control arms:

This control group will be treated as the open-placebo group, but do not receive any placebos. The purpose of this control group is to match for time with practioner and to assess general improvements of allergic symptoms due to changes in weather (pollen) conditions.

Description of the detailed information on placebo arms:

After signing the informed consent, patients will be informed about the power of placebos. They will be told that placebos are inactive substances and that they contain no medications. Although placebos contain no medication, placebo effects may still be powerful. The effect will be explained to them by pointing out that the body may automatically respond to taking placebo pills, like Pavlov’s dogs that salivated when they heard the bell. In addition, they will be told that a positive attitude may be helpful for the placebo effect, but is not necessary. Last, they will be instructed that those participants who will be in the placebo group need to take the placebos faithfully. These four statements are identical to the instruction used in previous studies on open-placebos. The further treatment is outlined above.

Description of the detailed information on placebo arms:

Same as before, but here the description of the power of placebos (as outlined above) will be omitted.

Randomization: Will be done by using sealed envelopes indicating the group assignment.

**F. Data Analysis**

Outcome Measures: Two outcome measures will be used at baseline and endpoint of this study:

Allergic symptoms questionnaire:

Participants will be asked to assess their allergic symptoms by using a questionnaire describing different symptoms (itching, prickling, or burning feelings in the nose; constipated or running nose; impaired sense of smell or taste; sneezing fits; feeling like having a cold; itching or irritated skin; eczema on the skin; itching, burning or red eyes; billowed eyelids; itching, prickling or scratching feelings in the throat; sore throat; burning feeling or mucus in the mouth or throat; billowed mucosa; breathlessness; cough; headaches; feelings of exhaustion; lack of concentration; tiredness; disorders of the gastrointestinal tract). The patients will indicate their response on a seven-point scale ranging from ‘never’ (1) to ‘always’ (7). For further analysis we will calculate a composite score including all symptoms (see Schaefer et al., 2016). This is the primary outcome parameter.

Quality of Life (SF36): The SF-36 is a German version of the health survey developed by Ware and Sherbourne (1992) in order to examine subjective well-being and quality of life. This instrument assesses the quality of life with respect to the perception of the health both for patients and healthy people. It includes one multi-item scale that assesses different health concepts such as limitations in physical activities because of health problems, limitations in social activities because of physical or emotional problems, limitations in usual role activities because of physical health problems, bodily pain, general mental health (psychological distress and well-being), limitations in usual role activities because of emotional problems, vitality (energy and fatigue), and general health perceptions.

Planned Statistical Analyses

We will conduct analyses of variance (ANOVA) with three factors (time, placebos, information on the power of placebos) for allergic symptoms as well as for quality of life scores. In addition, we will test if baseline scores are different. All tests will be two-tailed with alpha set at .05.

Statistical Power

Based on effect sizes of our previous study (Schaefer et al., 2016), we will need 104 participants for an alpha set at .05. However, given that this is a pilot study and data collection is limited due to weather (pollen) conditions, we aim to include 60 patients in this study.

**G. Subject Selection**

Participants were recruited from fliers in the local university and via social media.

Inclusion criteria:

1. Age between 18-60 years

2. Allergic rhinitis diagnosed by a physician

3. Taking medication against allergic rhinitis

Exclusion criteria:

1. Pregnancy

2. Diabetes

3. Psychiatric or neurological diseases

**H. Possible Benefits**

Open placebos may or may not help the patients. The patient’s participation may help researchers develop more effective medications.

**I. Possible Risks**

Since no active treatment is being given and we ask the patients not to change the usual medications, there is a minimal risk to the patient.

**J. Data Location**

Study visits will be at the Medical School Berlin, Berlin, Germany.

**K. Data Security**

Data will kept in a locked room, with only researchers listed above have access to. Data will be treated according to the rules of the ethical committee which has approved this study.
