# Supplementary material for: Why do open-label placebos work? A randomized controlled trial of an open-label placebo induction with and without extended information about the placebo effect in allergic rhinitis
Source: PLoS One. 2018 Mar 7;13(3):e0192758. doi: 10.1371/journal.pone.0192758 (PMC5841659; doi:10.1371/journal.pone.0192758)
Supplement: S1 Study Protocol for Ethical Committee — (PDF) [file pone.0192758.s004.pdf]

Antrag auf Stellungnahme der Ethikkommission der DGPs

1. Bezeichnung des Forschungsvorhabens

Embodiment und Moral: Dimensionen des Macbeth-Effekts

2. Name und Kontaktdaten des Antragstellers (Dienstanschrift):

Prof. Dr. Michael Schaefer
Medical School Berlin
Calandrellistr. 1-9
12247 Berlin
Fon: +49 30 / 76 68 37 53-63
Fax: +49 30 / 76 68 37 53-69
michael.schaefer@medicalschool-berlin.de

3. Angaben zu den Rahmenbedingungen des Vorhabens

Es handelt sich um einen Antrag auf Finanzierung durch die DFG. Eine Stellungnahme der Ethikkommission wird verlangt. Der Antrag ist in Vorbereitung.

4. Gegenstand und Verfahren des Vorhabens

Gegenstand. Neuere Studien zeigen, dass abstrakte Kognitionen offenbar nicht unabhängig von der körperlichen Erfahrungswelt sind. Diese enge Wechselwirkung zwischen Körper und Geist wird als Embodiment bezeichnet. Ein bemerkenswertes Beispiel ist der Macbeth-Effekt, der eine enge Verbindung zwischen physiologischer Sauberkeit und moralischer Reinheit beschreibt. So konnte gezeigt werden, dass ein moralisch schlechtes Verhalten später zu einer attraktiveren Bewertung von Seifenprodukten führt (im Vergleich zu einer moralisch korrektem Handlungsweise und zu anderen Waren). Und umgekehrt: Allein daran zu denken, den eigenen Körper zu reinigen, lässt nachfolgende Beurteilungen unmoralischer Handlungen milder ausfallen. Demnach können auch höhere Kognitionen wie moralische Beurteilungen von körperlichen Aspekten beeinflusst werden. Die beabsichtigten Studien wollen den Macbeth-Effekt näher beleuchten. So soll der Macbeth-Effekt bei Kindern verschiedener Altersgruppen sowie bei Patienten mit Angststörungen untersucht werden. Darüber hinaus soll der Macbeth-Effekt auch bei einer Gruppe von Allergie-Patienten untersucht werden. Allergiepatienten sind bekannt für eine hohe Aufmerksamkeit bzgl. Ihres Körpers. Wir vermuten entsprechend eine starke Ausprägung der Reinlichkeitsmetapher, also des Macbeth Effekts. Schließlich sollen die neuronalen Korrelate des Effektes mit der funktionellen Kernspintomographie weiter erforscht werden. Wir erhoffen uns von den Ergebnissen ein besseres Verständnis dieses bemerkenswerten Effektes.

Methoden. *Verhaltensstudien (Studie 1 - 3), fMRT-Studie (Studie 4).*

Experimentelle Aufgaben.

In allen 4 Studien soll der Macbeth-Effekt untersucht werden, d.Entscheidungs-. Probanden sollen an ein unmoralisches (bzw. moralisches) Verhalten denken (Priming) und danach Seifen- und andere Produkte (Schokoladenriegel, etc.) hinsichtlich ihrer Attraktivität einschätzen. Die Stimuli sind dabei in anderen Studien vorab getestet und in ihrer Attraktivität als vergleichbar eingeschätzt worden (Zhong und Liljenquist, Science 2006; Denke et al., Cerebral Cortex 2016; Schaefer et al., SciRep 2015). Wir verwenden dabei die identischen Produkte wie in unseren Vorläuferstudien (Denke et al., Schaefer et al.). Allerdings müssen für Studie 2 neue Produkte getestet werden, die in dieser Altersgruppe als grundsätzlich vergleichbar attraktiv eingeschätzt werden.

Studie 1 will den Effekt bei Patienten mit generalisierter Angststörung untersuchen (ICD-10 F41.1). Die (nicht-stationären) Patienten sollen entweder eine unmoralische oder eine moralische Geschichte abschreiben. Danach sollen Seifen und andere Produkte hinsichtlich ihrer Attraktivität eingeschätzt werden.

Studie 2 will den Effekt bei Kindern unterschiedlicher Altersgruppen testen. So sollen Schüler im Alter von 6, 9 und 12 Jahren untersucht werden. Die Kinder sollen sich mit einer moralischen bzw. unmorali-

schen Situation beschäftigen und dann eine Reihe von Gegenständen hinsichtlich ihrer Attraktivität einschätzen (Seifen- und Kontrollprodukte).

Studie 3 soll den Macbeth-Effekt bei Patienten mit allergischer Rhinitis (Heuschnupfen) testen. Patienten mit allergischer Rhinitis weisen besonders starke Placeboeffekte auf (Czubalski und Zawisza, Acta Otolaryngol. 1976). So konnten wir zeigen, dass selbst eine offene, also wissentliche Gabe von Placebos die Symptome von allergischer Rhinitis signifikant reduzieren kann (Schaefer et al., submitted). Hier wollen wir derartige Patienten mit dem Macbeth-Effekt testen. Patienten mit allergischer Rhinitis sollen offen (unverschleiert) über zwei Wochen jeweils morgens und abends ein Placebo einnehmen. Das Placebo besteht dabei aus einer kleinen Zuckertablette. Eine Kontrollgruppe erhält kein Placebo. Alle Probanden nehmen weiter ihre bisherige Medikation ein. Darüber hinaus sollen alle Probanden über die gesamte Zeit ein Allergietagebuch führen, was unter anderem auch der Sicherung der Compliance der Kontrollgruppe dienen soll. Um auch den Einfluss einer ausführlichen Beschreibung der Wirkungsweise von Placebos auf den open-label Placebo-Effekt zu untersuchen, sollen die Hälfte der Probanden darüber informiert werden, dass auch Placebos wirken können, während die andere Hälfte der Probanden diese ausführlichen Informationen nicht erhält. Nach zwei Wochen wollen wir erneut die Allergie-Symptome sowie den Macbeth-Effekt untersuchen. Es wird angenommen, dass es bei der Placebogruppe zu einer Symptomreduktion kommt (Schaefer et al., submitted). Wir nehmen weiter an, dass bei dieser Gruppe ein stärkerer Macbeth-Effekt zu beobachten ist. Dieser soll wieder gemessen werden durch Abschreiben eines (un)moralischen Textes und anschließendem Beurteilen der Attraktivität von Seifen- und Kontrollprodukten.

Studie 4 schließlich will die neuronalen Korrelate des Macbeth-Effektes untersuchen. Dazu werden Probanden im Kernspintomographen (fMRT) getestet. Im Scanner werden den Probanden kurze Szenarien präsentiert, bei denen die Probanden sich in die Protagonisten hineinversetzen sollen. Bei der Hälfte der Szenarien sollen die Probanden sich dabei in einen Protagonisten hineinversetzen, der sich in diesem Szenario unmoralisch verhält, z.B. einem hilfebedürftigen Kollegen nicht hilft. Bei der anderen Hälfte sollen die Probanden sich in einen Protagonisten hineinversetzen, der sich moralisch verhält, z.B. einem hilfsbedürftigen Kollegen beisteht. Ein entsprechendes Szenario ist nachfolgend wiedergegeben (siehe auch Denke et al., 2016; Schaefer et al., 2015; Lee und Schwarz, 2010):

„Stellen Sie sich vor, Sie arbeiteten in einer großen Rechtsanwaltskanzlei und würden vor allem mit Ihrem Kollegen Sven konkurrieren. Heute morgen fanden Sie zufällig ein wichtiges Dokument auf dem Boden, welches offensichtlich Sven verloren hatte. Dieses Dokument ist für Sven von großer Bedeutung, könnte aber möglicherweise Ihrer eigenen Karriere nicht förderlich sein.“

Nach einigen Sekunden erscheint dann die folgenden Aufforderung:

„Hinterlassen Sie eine Nachricht auf Svens Mailbox. Sagen Sie ihm, wer Sie sind und dass sie das Dokument leider auch nicht gefunden haben!“ (unmoralische Bedingung).

Alternativ erscheint:

„Hinterlassen Sie eine Nachricht auf Svens Mailbox. Sagen Sie ihm, wer Sie sind und dass sie das Dokument gefunden haben!“ (moralische Bedingung).

Anschließend bekommen die Probanden dann wieder Bilder von Seifen und anderen Produkten gezeigt, die sie hinsichtlich der Attraktivität einschätzen sollen. Wir gehen dabei von aus, dass der Macbeth-

Effekt auf Hirnarealen beruht, denen auch im Zusammenhang der Spiegelneuronentheorie tragende Rollen zugeschrieben werden.

Durchführung.

Studie 1 und 2 sind reine Verhaltensstudien.

Studie 3 beinhaltet die Gabe von Placebos (hier Zuckerpillen), die die Probanden über zwei Wochen einnehmen sollen. Zu Beginn der Studie sollen die Probanden Fragebögen hinsichtlich ihrer allergischen Belastung sowie ihrer gesundheitlichen Lebensqualität beantworten. Die Hälfte der Probanden bekommt dann ausführliche Informationen zu Placebos. Dabei wird darüber informiert, dass Placebos wirkstofflose Scheinpräparate sind, aber viele Studien gezeigt haben, dass diese dennoch eine Wirkung haben können. Die Probanden werden dann erneut in Gruppen aufgeteilt. In der Placebogruppe sollen die Probanden offen (unverschleiert) über zwei Wochen jeweils morgens und abends ein Placebo einnehmen. In der Kontrollgruppe bekommen die Probanden kein Medikament. Alle Teilnehmer sollen ihre bisherige Medikation unverändert weiter einnehmen. Darüber hinaus sollen alle Probanden ein Tagebuch über ihre allergische Belastung führen. Nach zwei Wochen werden erneut allergische Belastung und gesundheitliche Lebensqualität gemessen. Bei diesem zweiten Termin wird schließlich auch für alle Probanden der Macbeth-Effekt gemessen (siehe oben).

Studie 4 ist eine fMRT-Studie.

Körperliche Beanspruchung. Keine.

Mentale Beanspruchung. Keine.

Preisgabe persönlicher Informationen. Keine persönlichen Informationen.

Täuschung und Aufklärung. Keine Täuschung. Am Ende der Experimente werden alle über den Zweck der Studien aufgeklärt.

5. Angaben zu Aufzeichnung, Aufbereitung, Speicherung und Löschung der Daten

Personenbezogene Daten.

Studien 1 und 2: Alter, Geschlecht und die Fragebogenergebnisse werden gespeichert. Studie 3: Wie 1, nur werden hier auch die Daten der Allergiesymptome gespeichert. Studie 4: Im Rahmen der Studie werden MRT-Daten der Probanden gespeichert.

Datenschutz. Kodierliste und persönliches Codewort.

Alle Daten werden pseudonymisiert und anschließend anonymisiert gespeichert. Die Zuordnung der Daten erfolgt über eine Kodierliste, die nur dem Studienleiter zugänglich ist. Die Kodierliste wird nur einmal in Papierform vorliegen, sie wird unter Verschluss gehalten und ist nur dem Studienleiter zugänglich.

Löschung der Daten.

Die Kodierliste mit den Namen der Probanden wird nach 5 Jahren vernichtet, es erfolgt eine Anonymisierung ohne Kenntnis des Probanden.

6. Gewinnung der Personenstichprobe und Vergütung von Probanden

Rekrutierung.

In Studie 1 werden Patienten über ambulante Träger rekrutiert (über Flyer und Aushänge). Konkret sollen in niedergelassenen Arztpraxen Flyer verteilt werden, die auf die Studie aufmerksam machen.

Studie 2 sucht Probanden über lokale Grundschulen.

Probanden der Studien 3 und 4 werden über Aushänge in den lokalen Universitäten sowie über soziale Medien rekrutiert. Da eine hohe Anzahl von Menschen an Heuschnupfen leidet, sind wir zuversichtlich, über diese konventionellen Wege entsprechende Probanden zu bekommen (Studie 3).

Personenstichprobe aus Datenbank? Entfällt.

Merkmale der Personenstichprobe.

Studie 1: Es werden Patienten im Altern von 18-60 Jahren (ca. 50% weiblich) mit einer diagnostizierten generalisierten Angststörung gesucht, die sich in einer ambulanten Behandlung befinden.

Studie 2: Es werden Schüler im Alter von 6, 9 und 12 Jahren gesucht, ca. 50% weiblich.

Studie 3: Es werden Patienten im Alter von 18-60 Jahren gesucht (ca. 50% weiblich), die unter einer allergischen Rhinitis (Heuschnupfen) leiden.

Studie 4: Es werden Probanden im Alter von 18-60 Jahren, ca. 50% weiblich, gesucht.

Einschluss- und Ausschlusskriterien.

Einschlusskriterien Studie 1: Alter 18-60 Jahre, ärztlich diagnostizierte generalisierte Angststörung (ICD-10 F41.1)
Ausschlusskriterien: ärztliche diagnostizierte Depression

Einschlusskriterien Studie 2: Alter 6 bis 12 Jahre, Zustimmung der Erziehungsberechtigten
Ausschlusskriterien: keine

Einschlusskriterien Studie 3: Alter 18-60 Jahre, ärztlich diagnostizierte allergische Rhinitis
Ausschlusskriterien: Diabetes, Schwangerschaft, psychiatrische oder neurologische Vorerkrankungen

Einschlusskriterien Studie 4: Alter 18-60 Jahre
Ausschlusskriterien: Metall im Körper, Platzangst, Tinnitus (siehe MRT-Fragebogen in Einverständniserklärung zur Studie 4).

Internetbasierte Datengewinnung. Entfällt.

Teilnahmevergütung.

Studie 1 und 2: Die Probanden erhalten keine Vergütung. Studie 3: Die Probanden erhalten 3 Versuchspersonenstunden. Studie 4: Die Probanden erhalten 3 Versuchspersonenstunden.

7. Freiwilligkeit der Teilnahme und Rücktritt

Freiwilligkeit.

Alle Teilnehmer werden in der Teilnehmerinformation auf Freiwilligkeit der Teilnahme hingewiesen.

Rücktritt.

Alle Teilnehmer werden in der Teilnehmerinformation auf eine jederzeitige Rücktrittsmöglichkeit hingewiesen, die mit keinerlei Nachteilen verbunden ist. Auch auf die Löschung der eigenen Daten bis zum Zeitpunkt ihrer Anonymisierung wird hingewiesen.

8. Umgang mit auffälligen Befunden

Aufklärung.

Betrifft nur Studie 4: Probanden werden darauf hingewiesen, dass Sie über zufällige Befunde im Rahmen der fMRT-Studie benachrichtigt werden (siehe Teilnahmeinformation).

Teilnahmebeschränkung.

Nur Studie 4: In der Teilnehmerinformation wird darauf hingewiesen, dass man nur teilnehmen kann, wenn man der Mitteilung von auffälligen Befunden zustimmt. Der Proband unterschreibt diese Zustimmung in der Einverständniserklärung.

9. Informiertheit und Einwilligung

Informiertheit.

Probanden werden vollständig über die Experimente informiert.

Einwilligung.

Teilnehmerinformation und Einwilligung sind angefügt.

Bild- und Tonaufnahmen.

Entfällt.

10. Bestätigung

Ich bestätige, dass an meiner Universität keine lokale Ethikkommission eingerichtet ist, die meinen Antrag entgegen nehmen kann.

x ja ❑ nein *(bitte Zutreffendes ankreuzen)*

Berlin, den 30.04.16	Michael Schaefer
Ort, Datum	Unterschrift Antragsteller

Medical School Berlin
Prof. Dr. habil. Michael Schaefer

Prof. Dr. habil. Michael Schaefer
Medical School Berlin
Calandrellistr. 1-9
12447 Berlin, Germany
Tel. : 030 / 76 68 37 53-63
michael.schaefer@medicalschool-berlin.de

Allgemeine Teilnehmerinformation über eine Untersuchung der Medical School Berlin

Titel der Studie: Entscheidungs- und Beurteilungsverhalten in verschiedenen Situationen

[für Studie 1:]

Herzlich willkommen bei unserer Studie zum "Entscheidungs- und Beurteilungsverhalten in verschiedenen Situationen"! Wir danken Ihnen für Ihr Interesse an dieser Studie.

Wir untersuchen mit dieser Studie, wie Personen Entscheidungsaufgaben durchführen. In diesem Experiment sollen Sie einen Text abschreiben und anschließend Beurteilungsaufgaben durchführen.

Ablauf der Studie

Das folgende Experiment dauert ca. 15 Minuten. Ihre Aufgabe ist es, einen Text abzuschreiben und danach einige Fragebögen zu beantworten.

Sollten Sie noch Fragen haben, wenden Sie sich damit bitte an den Versuchsleiter.

[Für Studie 2:]

Herzlich willkommen bei unserer Studie "Entscheidungs- und Beurteilungsverhalten in verschiedenen Situationen"! Wir danken Ihnen für Ihr Interesse an dieser Studie.

Wir untersuchen mit dieser Studie Entscheidungs- und Beurteilungsverhalten bei Kindern unterschiedlicher Altersklassen. Wir würden uns freuen, wenn Sie ihrem Kind die Teilnahme hieran ermöglichen würden.

Ablauf der Studie

Das Experiment besteht aus Fragen zum Erleben von Regeln im Alltag und einem Beurteilungsexperiment. Insgesamt dauert das Experiment ca. 15 min. Wir werden ihr Kind nach einer Situation fragen, in der es erlebt hat, wie sich an Regeln gehalten wurde oder wie sich nicht an Regeln gehalten wurde. Danach soll ihr Kind eine Reihe von Gegenständen einschätzen, inwiefern es diese mag.

Sollten Sie noch Fragen haben, können Sie sich gerne an uns wenden.

[Für Studie 3:]

Herzlich willkommen bei unserer Studie zu "Entscheidungs- und Beurteilungsverhalten in verschiedenen Situationen”. Wir danken Ihnen für Ihr Interesse an dieser Studie.

Wir untersuchen mit dieser Studie psychophysische Interaktionen sowie Entscheidungsaufgaben und Verhalten bei Patienten mit allergischer Rhinitis (Heuschnupfen).

Ablauf der Studie

Die Studie dauert insgesamt etwa 2 Wochen und ist mit zwei Terminen für die Erhebungen verbunden.

Zu Beginn der Studie erfassen wir den Ausgangsstatus Ihrer Allergie und Ihres Wohlbefindens. Anschließend erklären wir Ihnen dann ein Allergietagebuch, das wir Sie bitten, über den gesamten Zeitraum von 14 Tagen zu führen. Hierin sollen sie täglich am Abend angeben, wie stark die allergische Belastung für Sie war. Es ist wichtig, dass Sie dieses Tagebuch jeden Abend regelmäßig ausfüllen.

Darüber hinaus werden einige Teilnehmer ein wirkungsloses Scheinpräparat („Placebo“) bekommen. Das Placebo hat keinerlei Wirkstoffe und ähnelt in seiner Zusammensetzung einer Zuckerpille. Es soll über zwei Wochen jeweils morgens und abends eingenommen werden.

Nach Abschluss der Studie erheben wir erneut den Status Ihrer Allergie sowie Ihr aktuelles Wohlbefinden. Darüber hinaus führen wir an diesem Termin noch Aufgaben durch, in denen Sie bestimmte Gegenstände beurteilen sollen.

Sollten Sie noch Fragen haben, wenden Sie sich damit bitte an den Versuchsleiter.

Freiwilligkeit und Anonymität

Die Teilnahme an der Studie ist freiwillig. Sie können jederzeit und ohne Angabe von Gründen die Teilnahme an dieser Studie beenden, ohne dass Ihnen daraus Nachteile entstehen.

Die im Rahmen dieser Studie erhobenen, oben beschriebenen Daten und persönlichen Mitteilungen werden vertraulich behandelt. So unterliegen diejenigen Projektmitarbeiter, die durch direkten Kontakt mit Ihnen über personenbezogene Daten verfügen, der Schweigepflicht. Des weiteren wird die Veröffentlichung der Ergebnisse der Studie in anonymisierter Form erfolgen, d. Entscheidungs-. ohne dass Ihre Daten Ihrer Person zugeordnet werden können.

Datenschutz

Die Erhebung und Verarbeitung Ihrer oben beschriebenen persönlichen Daten erfolgt pseudonymisiert in der Medical School Berlin unter Verwendung einer Nummer und ohne Angabe Ihres Namens. Es existiert eine Kodierliste auf Papier, die Ihren Namen mit der Nummer verbindet. Die Kodierliste ist nur den Versuchsleitern und dem Projektleiter zugänglich; das heißt, nur diese Personen können die erhobenen Daten mit meinem Namen in Verbindung bringen. Die Kodierliste wird in einem abschließbaren Schrank aufbewahrt und nach Abschluss der Datenauswertung, spätestens aber am 31.12.2017, vernichtet. Ihre Daten sind dann anonymisiert. Damit ist es niemandem mehr möglich, die erhobenen Daten mit Ihrem Namen in Verbindung zu bringen. Die anonymisierten Daten werden mindestens 10 Jahre gespeichert. Solange die Kodierliste existiert, können Sie die Löschung aller von Ihnen erhobenen Daten verlangen.

Ist die Kodierliste aber erst einmal gelöscht, können wir Ihren Datensatz nicht mehr identifizieren. Deshalb können wir Ihrem Verlangen nach Löschung Ihrer Daten nur solange nachkommen, wie die Kodierliste existiert.

[Studie 3] Versuchspersonenstunden

Für die Teilnahme an diesem Experiment erhalten Sie 3 Versuchspersonenstunden.

Haben Sie noch Fragen? Bitte sprechen Sie uns an!

Vielen Dank für Ihr Interesse!

Medical School Berlin
Prof. Dr. habil. Michael Schaefer

Prof. Dr. habil. Michael Schaefer
Medical School Berlin
Calandrellistr. 1-9
12447 Berlin, Germany
Tel. : 030 / 76 68 37 53-63
michael.schaefer@medicalschool-berlin.de

[Studie 1,2 und 3:] Einwilligungserklärung

Medical School Berlin

Titel der Studie: Entscheidungs- und Beurteilungsverhalten in verschiedenen Situationen

Ich (Name des Teilnehmers /der Teilnehmerin in Blockschrift)

____

bin schriftlich über die Studie und den Versuchsablauf aufgeklärt worden. [Studie 1:] Ich willige ein, an dem in der Teilnehmerinformation beschriebenen Experiment teilzunehmen. [Studie 2]: Ich willige ein, dass mein Kind an dem in der Teilnehmerinformation beschriebenen Experiment teilnimmt. [Studie 3:] Ich willige ein, an dem in der Teilnehmerinformation beschriebenen Experiment teilzunehmen.

Sofern ich Fragen zu dieser vorgesehenen Studie hatte, wurden sie von Herrn/Frau _________________________ vollständig und zu meiner Zufriedenheit beantwortet.

Mit der beschriebenen Erhebung und Verarbeitung der Daten (den Ergebnissen aus den Fragebögen) bin ich einverstanden. Die Aufzeichnung und Auswertung dieser Daten erfolgt pseudonymisiert an der Medical School Berlin, unter Verwendung einer Nummer und ohne Angabe meines Namens [für Studie 2: „....des Namens des Kindes“, analog auch in nachfolgenden Textstellen]. Es existiert eine Kodierliste auf Papier, die meinen Namen mit dieser Nummer verbindet. Diese Kodierliste ist nur den Versuchsleitern und dem Projektleiter zugänglich, das heißt, nur diese Personen können die erhobenen Daten mit meinem Namen in Verbindung bringen. Nach Abschluss der Datenauswertung, spätestens am 31.12.2017, wird die Kodierliste gelöscht. Meine Daten sind dann anonymisiert. Damit ist es niemandem mehr möglich, die erhobenen Daten mit meinem Namen in Verbindung zu bringen. Mir ist bekannt, dass ich mein Einverständnis zur Aufbewahrung bzw. Speicherung dieser Daten widerrufen kann, ohne dass mir daraus Nachteile entstehen. Ich bin darüber informiert worden, dass ich jederzeit eine Löschung all meiner Daten verlangen kann. Wenn allerdings die Kodierliste bereits gelöscht ist, kann mein Datensatz nicht

mehr identifiziert und also auch nicht mehr gelöscht werden. Meine Daten sind dann anonymisiert. Ich bin einverstanden, dass meine anonymisierten Daten zu Forschungszwecken weiter verwendet werden können und mindestens 10 Jahre gespeichert bleiben.

Ich hatte genügend Zeit für eine Entscheidung und bin bereit, an der o.g. Studie teilzunehmen. Ich weiß, dass die Teilnahme an der Studie freiwillig ist und ich die Teilnahme jederzeit ohne Angaben von Gründen beenden kann.

Eine Ausfertigung der Teilnehmerinformation über die Untersuchung und eine Ausfertigung der Einwilligungserklärung habe ich erhalten. Die Teilnehmerinformation ist Teil dieser Einwilligungserklärung.

Ort, Datum & Unterschrift des Teilnehmers: Name des Teilnehmers in Druckschrift:

_______________________________ _______________________________

[nur für Studie 2:

Ort, Datum & Unterschrift des Erziehungsberechtigten des Teilnehmers: Name des Teilnehmers in Druckschrift:

_______________________________ _______________________________]

Ort, Datum & Unterschrift des Versuchsleiters: Name des Versuchsleiters in Druckschrift:

_______________________________ _______________________________

Zusatzvereinbarung für künftige Kontaktaufnahmen im Rahmen dieser Studie

Ich gebe mein Einverständnis, dass im Falle einer Fortführung dieser Studie oder von Anschlussstudien die Kodierliste weiterhin für die Dauer von fünf Jahren aufbewahrt wird und ich für Anschlussstudien kontaktiert werden darf. Mir ist bekannt, dass ich mein Einverständnis zur Aufbewahrung bzw. Speicherung dieser Daten widerrufen kann, ohne dass mir daraus Nachteile entstehen. Ich bin darüber informiert worden, dass ich jederzeit eine Löschung all meiner Daten verlangen kann. Wenn allerdings die Kodierliste bereits gelöscht ist, kann mein Datensatz nicht mehr identifiziert und also auch nicht mehr gelöscht werden.

O JA O NEIN.

Rückmeldung von Ergebnissen

Ich bin daran interessiert, etwas über die grundsätzlichen Ergebnisse der Studie zu erfahren, und bitte hierzu um Übersendung entsprechender Informationen.

O JA O NEIN.

Ort, Datum & Unterschrift des Teilnehmers: ______________________

Name des Teilnehmers in Druckschrift: ______________________

Bei Fragen oder anderen Anliegen kann ich mich an folgende Personen wenden:

Versuchsleiter:	Projektleiter:
N.N.	*Prof. Dr. Michael Schaefer*
>Anschrift<	*Medical School Berlin, Calandrellistr. 1-9, 12447 Berlin*
>Telefonnummer<	*030 / 76 68 37 53-63*
>E-Mail Adresse<	*michael.schaefer@medicalschool-berlin.de*

Medical School Berlin
Prof. Dr. habil. Michael Schaefer

Prof. Dr. habil. Michael Schaefer
Medical School Berlin
Calandrellistr. 1-9
12447 Berlin, Germany
Tel. : 030 / 76 68 37 53-63
michael.schaefer@medicalschool-berlin.de

[Studie 4:] Teilnehmerinformation für MRT-Studie an der Medical School Berlin

Titel der Studie: Entscheidungs- und Beurteilungsverhalten in verschiedenen Situationen

Liebe Teilnehmerin, lieber Teilnehmer,

im Rahmen einer wissenschaftlichen Studie möchten wir bei Ihnen eine Magnetresonanztomographie (MRT; Kernspintomographie) durchführen. In den folgenden Abschnitten erfahren Sie Näheres über diese Untersuchung. Fragen Sie uns gerne, wenn Sie etwas nicht verstanden haben oder mehr über die Untersuchungsmethode erfahren möchten.

Zweck und Ablauf der Untersuchung

Die Untersuchung dient der Erforschung der Funktionsweise des menschlichen Gehirns, wobei hier vor allem neuronale Korrelate von Entscheidungsverhalten untersucht werden sollen.

Die MRT-Technologie ist ein sogenanntes nicht-invasives Verfahren, d. Entscheidungs-. es ist für den Körper nach heutigem Erkenntnisstand unschädlich. Im Unterschied zu anderen diagnostischen Verfahren wird bei der MRT-Technologie keine ionisierende Strahlung (Radioaktivität) eingesetzt. Nach heutigem Wissensstand, basierend auf mehr als 20-jähriger Erfahrung mit der MRT-Technologie, die täglich in allen größeren Kliniken eingesetzt wird, sind keine Nebeneffekte bekannt. Darüber hinaus gibt es keine Hinweise auf negative Langzeiteffekte der MRT-Technologie auf den menschlichen Körper.

Sie werden auf einem Tisch liegen, welcher Sie in die zylinderförmige Öffnung des MR-Tomographen hineinfährt, wo sich die starken Magnetfelder befinden. Zusätzlich wird ein Rahmen (die Magnetspule) um Ihren Kopf gelegt. Während der Messung werden Sie ein Klopfen hören. Um Schäden am Gehör zu vermeiden, werden Sie vor der Messung einen Gehörschutz erhalten. Die Untersuchungszeit liegt bei ca. 60 Minuten. Sie werden gebeten, einfache Entscheidungsaufgaben im MR-Tomographen durchzuführen

(Dauer ca. 45 min). Davor erfolgt noch eine genauere Aufnahme von der Struktur Ihres Gehirns (Dauer ca. 5 min). Vor der Untersuchung ist deshalb ein Gang zur Toilette ratsam. Sie haben während der Untersuchung jederzeit die Möglichkeit, mit den Untersuchern über eine Wechselsprechanlage in Kontakt zu treten. Zusätzlich bekommen Sie einen Alarmknopf (Druckball) mit in den MR-Tomographen. Auf Ihren Wunsch hin können Sie jederzeit aus dem MR-Tomographen hinausgefahren werden. Abgesehen von möglichen Unbequemlichkeiten, die vom langen, stillen Liegen resultieren, sollten Sie keine Beschwerden während der Untersuchung haben.

Die Anwendung von Magnetfeldern bei der MRT-Untersuchung schließt die Teilnahme von Personen aus, die elektrische Geräte (z. B. Herzschrittmacher, Medikamentenpumpen usw.) oder Metallteile (z. B. Schrauben nach Knochenbruch) im oder am Körper haben. Ebenso ist die Teilnahme von Frauen im gebärfähigen Alter, die schwanger sind oder schwanger sein könnten, ausgeschlossen, da die Wirkung des Magnetfeldes auf den Embryo nicht ausreichend untersucht ist. Die räumlichen Verhältnisse im MR-Tomographen lassen es nicht zu, Personen mit starken Rückenbeschwerden oder übermäßigem Übergewicht zu untersuchen. Auch sollten große, schnelle Bewegungen im MR-Tomographen unterbleiben, um keinen Magnetstrom zu induzieren.

Auffällige Befunde

Die Untersuchung dient ausschließlich Forschungszwecken. Eine medizinische oder psychologische Beurteilung Ihrer Daten erfolgt nicht. Es könnte uns jedoch ein ungewöhnliches Untersuchungsergebnis auffallen. In diesem Fall werden wir Sie darüber informieren und Ihnen empfehlen, dieses Ergebnis bei Ihrem Hausarzt diagnostisch weiter abklären zu lassen. Nur wenn Sie damit einverstanden sind, dass wir Sie ggf. über einen auffälligen Befund informieren, können Sie an dieser Studie teilnehmen. Sofern bei dieser diagnostischen Abklärung eine Erkrankung festgestellt werden sollte, könnten Ihnen daraus unter Umständen Nachteile entstehen, z. B. der Abschluss einer privaten Krankenversicherung oder einer Lebensversicherung erschwert werden.

Versuchspersonenstunden

Für die Teilnahme an diesem fMRT-Experiment erhalten Sie 3 Versuchspersonenstunden gutgeschrieben.

Medical School Berlin
Prof. Dr. habil. Michael Schaefer

MSB Medical School Berlin
Hochschule für Gesundheit und Medizin

Prof. Dr. habil. Michael Schaefer
Medical School Berlin
Calandrellistr. 1-9
12447 Berlin, Germany
Tel. : 030 / 76 68 37 53-63
michael.schaefer@medicalschool-berlin.de

[Studie 4:] Fragebogen für die Teilnahme an MRT-Studien

Fragebogen I (vor der MRT-Untersuchung) Wir bemühen uns, die MRT-Untersuchung für Sie so wenig belastend wie möglich zu gestalten.

Dieser Fragebogen dient dazu, mögliche Risiken vor der MRT-Untersuchung abzuschätzen.

1. Hatten Sie Operationen? Wenn ja, wann und welche?
2. Für Frauen im gebährfähigen Alter:
    a. Sind Sie schwanger? [*ja, nein, weiß nicht*]
    Falls *ja*: Ausschluss. Sonst:
    b. Kann eine Schwangerschaft definitiv ausgeschlossen werden (z. B. wegen Sterilisation, Einnahme von Kontrazeptiva, sexueller Inaktivität seit letzter Regelblutung, etc.)? [*ja, nein*]
    Falls *ja*: weiter bei Punkt 3. Sonst:
    c. Wurde ein Schwangerschaftstest durchgeführt? [*ja*, *nein*]
    Falls *nein*: Ambulanten Schwangerschaftstest aushändigen. Sonst:
    d. War das Ergebnis positiv? [*ja*, *nein*]
    Falls *ja*: Ausschluss.
3. Tragen Sie einen Herzschrittmacher oder andere Stimulationsgeräte (künstliche Herzklappe, Metallprothesen, chirurgische Clips, Hörhilfen, Spirale)?
4. Haben Sie eine Medikamentenpumpe/ Infusionsgerät implantiert?
5. Haben Sie Metallteile im oder am Körper - insbesondere am Auge (chirurgische Schrauben oder Platten, Metallsplitter usw.)?

6. Benutzen Sie schwermetallhaltiges Make-up oder haben Sie Tätowierungen?
7. Haben Sie in der vergangenen Woche Schleifarbeiten oder ähnliches an metallischen Werkstoffen durchgeführt?
8. Leiden Sie an Klaustrophobie (Angst vor räumlicher Enge, "Platzangst")?
9. Leiden Sie an Rücken- oder Nackenschmerzen?
10. Haben Sie Kreislauf- oder Atembeschwerden (z. B. Hypertonie, Asthma bronchiale)?
11. Leiden Sie unter Bewusstlosigkeit, Epilepsie oder Krämpfen?

Metallteile, die in das Magnetfeld gelangen, können zu Verletzungen und Bildartefakten führen! Legen Sie deshalb alle metallenen oder magnetischen Gegenstände vor dem Betreten des Untersuchungsraumes ab: z. B. **Uhr, Brille, Schlüssel, (Piercing-)Schmuck, Haarnadeln/-spangen, Geldmünzen, Kreditkarten (Magnetstreifen wird gelöscht), Ohrschmuck, herausnehmbarer Zahnersatz, Zahnspange, Gürtelschnallen, BH mit Metallbügel, Nikotinpflaster.**

Fragebogen II (nach der MRT-Untersuchung)

Wir bemühen uns, die MRT-Untersuchung für Sie so wenig belastend wie möglich zu gestalten. Dieser Fragebogen hilft uns, mögliche Unannehmlichkeiten schnell zu erkennen und zu beseitigen.

1. Spürten Sie Unwohlsein?
2. Spürten Sie Angst (z. B. vor der Enge) ?
3. Hatten Sie Rücken- oder Nackenschmerzen?
4. Hatten Sie Schmerzen wegen des langen Liegens?
5. Spürten Sie unangenehme Wärme oder Hitze?
6. War die Lärmbelästigung erträglich?
7. War der Sprechkontakt zum Untersuchungspersonal einwandfrei?

Bemerkungen / Hinweise / Kommentare:

Medical School Berlin

Prof. Dr. habil. Michael Schaefer

Prof. Dr. habil. Michael Schaefer

Medical School Berlin

Calandrellistr. 1-9

12447 Berlin, Germany

Tel. : 030 / 76 68 37 53-63

michael.schaefer@medicalschool-berlin.de

[Studie 4:] Einwilligungserklärung

Medical School Berlin

Titel der Studie: Entscheidungs- und Beurteilungsverhalten in verschiedenen Situationen

Ich (Name des Teilnehmers /der Teilnehmerin in Blockschrift)

____

bin schriftlich über die Studie und den Versuchsablauf aufgeklärt worden. Ich willige ein, an dem in der Teilnehmerinformation beschriebenen Experiment im Kernspintomographen teilzunehmen.

Sofern ich Fragen zu dieser vorgesehenen Studie hatte, wurden sie von Herrn/Frau ________________________ vollständig und zu meiner Zufriedenheit beantwortet.

Mit der beschriebenen Erhebung und Verarbeitung der Daten bin ich einverstanden. Die Aufzeichnung und Auswertung dieser Daten erfolgt pseudonymisiert an der Medical School Berlin, unter Verwendung einer Nummer und ohne Angabe meines Namens. Es existiert eine Kodierliste auf Papier, die meinen Namen mit dieser Nummer verbindet. Diese Kodierliste ist nur den Versuchsleitern und dem Projektleiter zugänglich, das heißt, nur diese Personen können die erhobenen Daten mit meinem Namen in Verbindung bringen. Nach Abschluss der Datenauswertung, spätestens am 31.12.2017, wird die Kodierliste gelöscht. Meine Daten sind dann anonymisiert. Damit ist es niemandem mehr möglich, die erhobenen Daten mit meinem Namen in Verbindung zu bringen. Mir ist bekannt, dass ich mein Einverständnis zur Aufbewahrung bzw. Speicherung dieser Daten widerrufen kann, ohne dass mir daraus Nachteile entstehen. Ich bin darüber informiert worden, dass ich jederzeit eine Löschung all meiner Daten verlangen kann. Wenn allerdings die Kodierliste bereits gelöscht ist, kann mein Datensatz nicht mehr identifiziert und also auch nicht mehr gelöscht werden. Meine Daten sind dann anonymisiert. Ich bin einverstanden, dass meine anonymisierten Daten zu Forschungszwecken weiter verwendet werden können und mindestens 10 Jahre gespeichert bleiben.

Sollten behandlungsbedürftige Auffälligkeiten in den Hirnbildern aus der MRT-Untersuchung erkannt werden, bin ich damit einverstanden, dass mir diese mitgeteilt werden, so dass ich diese ggf. weiter abklären lassen kann. Ich wurde darüber informiert, dass die Information über auffällige Befunde u.U. mit versicherungsrechtlichen Konsequenzen verbunden sein kann.

Ich bin über Wesen, Bedeutung und Tragweite der geplanten MRT-Untersuchung aufgeklärt worden. Ich habe die Information zum Zweck der Untersuchung und die Teilnehmerinformation für MRT-Studien gelesen und willige in die Untersuchung ein. Zusätzlich bin ich ausführlich mündlich aufgeklärt und informiert worden. Ich habe weder Metallteile noch elektrische Geräte im Körper. Meine derzeitige körperliche Verfassung schließt eine Teilnahme an der MRT-Untersuchung nicht aus. Ich habe den Fragebogen für die Teilnahme an MRT-Studien wahrheitsgemäß ausgefüllt. Ich bin darauf aufmerksam gemacht worden, dass keine medizinische Diagnostik durchgeführt wird und die Hirnbilder auch nicht zur medizinischen Diagnostik verwendet werden. Für Frauen: Ich bin nicht schwanger. Eine Schwangerschaft kann entweder definitiv ausgeschlossen werden oder sie wird nach einer ordnungsgemäßen Anwendung eines Schwangerschaftstests nicht angezeigt.

Ich hatte genügend Zeit für eine Entscheidung und bin bereit, an der o.g. Studie teilzunehmen. Ich weiß, dass die Teilnahme an der Studie freiwillig ist und ich die Teilnahme jederzeit ohne Angaben von Gründen beenden kann. Ich weiß, dass ich in diesem Fall Anspruch auf Versuchspersonenstunden bzw. Vergütung für die bis dahin erbrachten Stunden habe.

Eine Ausfertigung der Teilnehmerinformation über die Untersuchung und eine Ausfertigung der Einwilligungserklärung habe ich erhalten. Die Teilnehmerinformation ist Teil dieser Einwilligungserklärung.

Ort, Datum & Unterschrift des Teilnehmers: ______________________

Name des Teilnehmers in Druckschrift: ______________________

Ort, Datum & Unterschrift des Versuchsleiters: ______________________

Name des Versuchsleiters in Druckschrift: ______________________

Zusatzvereinbarung für künftige Kontaktaufnahmen im Rahmen dieser Studie

Ich gebe mein Einverständnis, dass im Falle einer Fortführung dieser Studie oder von Anschlussstudien die Kodierliste weiterhin für die Dauer von fünf Jahren aufbewahrt wird und ich für Anschlussstudien kontaktiert werden darf. Mir ist bekannt, dass ich mein Einverständnis zur Aufbewahrung bzw. Speicherung dieser Daten widerrufen kann, ohne dass mir daraus Nachteile entstehen. Ich bin darüber informiert worden, dass ich jederzeit eine Löschung all meiner Daten verlangen kann. Wenn allerdings die Kodierliste bereits gelöscht ist, kann mein Datensatz nicht mehr identifiziert und also auch nicht mehr gelöscht werden.

O JA   O NEIN.

Rückmeldung von Ergebnissen

Ich bin daran interessiert, etwas über die grundsätzlichen Ergebnisse der Studie zu erfahren, und bitte hierzu um Übersendung entsprechender Informationen.

O JA   O NEIN.

Ort, Datum & Unterschrift des Teilnehmers: ______________________________

Name des Teilnehmers in Druckschrift: ______________________________

Bei Fragen oder anderen Anliegen kann ich mich an folgende Personen wenden:

Versuchsleiter:	Projektleiter:
N.N.	*Prof. Dr. Michael Schaefer*
>Anschrift<	*Medical School Berlin, Calandrellistr. 1-9, 12447 Berlin*
>Telefonnummer<	*030 / 76 68 37 53-63*
>E-Mail Adresse<	*michael.schaefer@medicalschool-berlin.de*
